# AlzPED: An Open Science Tool to Increase Rigor in Therapeutic Efficacy Testing in Alzheimer’s Animal Models

**DOI:** 10.1002/alz.087637

**Published:** 2025-01-09

**Authors:** Shreaya Chakroborty, Jaya Viswanathan, Maria Fe Lanfranco Gallofre, Zane Martin, Suzana S Petanceska, Lorenzo M Refolo

**Affiliations:** ^1^ National Institute on Aging, Bethesda, MD USA

## Abstract

**Background:**

Positive findings from testing therapeutics in AD animal models are often not translated to effective treatments due to the poor methodological rigor and inadequate reporting practices of therapeutic efficacy studies. The Alzheimer’s Disease Preclinical Efficacy Database (AlzPED), developed by the NIA, is a searchable and publicly available knowledgebase that prioritizes and promotes the use of rigorous methodology to ameliorate this translation gap. Through a checklist of experimental design elements – the Rigor Report Card ‐ AlzPED highlights reporting recommendations and standards while providing a practical tool to help plan rigorous therapeutic studies in animals. AlzPED also serves as a platform for creating citable preprints, including negative findings to mitigate the publication bias favoring positive reports.

**Methods:**

Using key word‐driven literature searches published studies are acquired and curated by two subject‐matter experts for bibliographic details, funding source, study goals and principal findings, data on relevant translational criteria like therapy type, therapeutic agent, therapeutic target, animal models, and AD‐related outcome measures. Rigor is assessed with the Rigor Report Card.

**Results:**

AlzPED hosts curated summaries from 1400 published preclinical therapeutic studies in AD animal models, and data related to 274 therapeutic targets, 1206 therapeutic agents, 226 animal models, more than 2500 AD‐related outcome measures, and thousands of principal findings. Evaluation of Rigor Report Cards from 1400 published preclinical therapeutic studies demonstrates significant under‐reporting of critical elements of methodology such as power/sample size calculation (4% of curated studies), blinding for treatment (10%), randomization (32%), balancing for sex (24%), inclusion/exclusion criteria (7%). All analytics are shared on AlzPED under the principles of open science.

**Conclusions:**

Rigorous experimental design and transparent reporting are essential to inform future research, science policies, and successful clinical trials. Adopting a standardized set of best practices like those proposed by AlzPED can improve the predictive power of preclinical studies in AD animal models and promote the effective translation of drug testing data to the clinic.

